# Zinc: A promising agent in dietary chemoprevention of cancer

**Published:** 2010-12

**Authors:** D. K. Dhawan, Vijayta D. Chadha

**Affiliations:** *Department of Biophysics, Panjab University, Chandigarh, India*

**Keywords:** Apoptosis, cancer, chemoprevention, essential elements, zinc, Zn deficiency

## Abstract

Proper intake of dietary nutrients is considered crucial for preventing the initiation of events leading to the development of carcinoma. Many dietary compounds have been considered to contribute in cancer prevention including zinc, which plays a pivotal role in host defense against the initiation and promotion of several malignancies. Zinc is an essential element that is integral to many proteins and transcription factors which regulate key cellular functions such as the response to oxidative stress, DNA replication, DNA damage repair, cell cycle progression, and apoptosis. Zinc has been ascribed roles in the metabolism and interaction of malignant cells, particularly in apoptosis. Zinc is involved in structural stabilization and activation of the p53 that appears to be an important component of the apoptotic process and also in activation of certain members of the caspase family of proteases. Zinc exerts a positive beneficial effect against chemically induced preneoplastic progression in rats and provides an effective dietary chemopreventive approach to disease in vulnerable section of population with family history of carcinoma. The present review provides an insight into the research conducted on animals as well as on human subjects for providing the concept that zinc deficiency is an important factor in the development and progression of malignancy and that zinc could be efficacious in the prevention and treatment of several cancers viz., colon, pancreas, oesophageal and head and neck. However, it needs further exploration with regard to other definitive bioassays including protein expression and documentation of specific molecular markers to establish the exact mechanism for zinc-mediated cancer chemoprevention. Preclinical trials need to investigate the genetic and epigenetic pathways of chemoprevention by zinc.

Numerous enzymes and proteins utilize first transition and Group IIB elements to carry out their biological functions and zinc is the most widely used of these elements in biology. Among the zinc enzymes are oxidoreductases, tranferases, hydrolases, lyases, isomerases and ligases. Indeed, zinc is the only metal encountered in each enzyme class. The notable selection and frequent utilization of zinc as the predominant functional element of so many biological molecules have been understood in terms of its chemical properties and its use in biochemical systems. Two properties of zinc need to be highlighted. First, unlike other metals, including those of IIB series, zinc is virtually nontoxic even at higher doses^1^. The homeostatic mechanisms that regulate its entry into, distribution in and excretion from cells and tissues are so efficient that no disorders are known to be associated with its excessive accumulation in contrast to iron, copper, mercury and other metals^2^. Second, its physical and chemical properties, including its generally stable association with macromolecules and its co-ordination flexibility, makes it highly adaptable to meet the needs of proteins and enzymes that carry out diverse biological functions^3^,^4^. These chemical properties form the basis for the extensive participation of zinc in protein, nucleic acid, carbohydrate and lipid metabolism as well as in the control of gene transcription and other fundamental biological processes.

Zinc is required in the diet of human beings in trace quantities, which is approximately 15 mg Zn/day^5^. It is found in all body tissues and fluids in relatively high concentrations, with 85 per cent of the whole body zinc in muscle and bone, 11 per cent in the skin and the liver and the remaining in all the other tissues^6^. The average amount of Zn in the adult body is about 1.4-2.3 g^6^. Zinc is present at higher concentrations in liver followed by pancreas, kidney, heart, pituitary, adrenal, and prostrate. There are many reports that clearly emphasize that zinc is a principal limiting factor in the nutrition of children and adolescents and that its deficiency probably accounted for the growth retardation so commonly seen in such age groups. Zinc is virtually nontoxic to living organisms. It is the only pre-, post-, and transitional element that is neither cytotoxic nor systematically toxic, nor is it carcinogenic, mutagenic, or teratogenic. Zinc is not stored in the body and excess intakes result in reduced absorption and increased excretion. However, there are reports on a few cases of acute Zn poisoning^7^. Although zinc is an essential element and is nontoxic at lower doses^1^, yet its metabolic role is not clearly known^8^. It has been considered a trace metal of prime concern as it is essential for carrying out function of various DNA and RNA synthesizing enzymes^9^. It is a part of most cellular aspects of body and participates in all major metabolic pathways and is involved in the development and maintenance of competent immune system.

Zinc deficiency, after prolonged reduction of intake or excessive uncompensated losses, has been described both in animals and humans. Long-term deprivation of Zn renders an organism more susceptible to injury induced by oxidative stress. More specifically, Zn deficiency increases the levels of lipid peroxidation in mitochondrial and microsomal membranes and the osmotic fragility of erythrocyte membranes, while the presence of Zn prevents lipid peroxidation and thus plays an important role in protecting the cells from oxidative stress^2^,^5^. Studies from our laboratory have also demonstrated the protective efficacy of zinc in regulating the activities of various antioxidant enzymes, thyroid hormones, liver marker enzymes as well as histological alterations under toxic conditions induced by various xenobiotics^10^–^15^.

## Zinc and cancer

A large body of evidence suggests that a significant percentage of deaths resulting from cancer could be avoided through greater attention to proper and adequate nutrition. Although many dietary compounds have been suggested to contribute in the prevention of cancer, yet there is a strong evidence to support the fact that zinc, a key constituent or cofactor of over 300 mammalian enzymes, may be of particular importance in host defense against the initiation and progression of cancer^16^. Remarkably, 10 per cent of the U.S. population consumes less than half the recommended dietary allowance for zinc and is at increased risk for zinc deficiency^16^. Zinc is known to be an essential component of DNA-binding proteins with zinc fingers, as well as copper/zinc superoxide dismutase and several proteins involved in DNA repair. Thus, zinc plays an important role in the functions of transcription factor, antioxidant defense system and DNA repair. Dietary deficiencies in the intake of zinc can contribute to single and double-strand DNA breaks and oxidative modifications to DNA that increase risk for cancer development^16^.

Zinc is an essential mineral that is integral to many enzymes and transcription factors that regulate key cellular functions such as the response to oxidative stress, DNA replication, DNA damage repair, cell cycle progression, and apoptosis. In particular, several proteins involved in DNA damage signaling and repair, replicative enzymes such as DNA and RNA polymerases and transcription factors such as tumour protein p53^17^, require zinc for proper functions^18^–^20^. Consequently, zinc deficiency could disrupt the function of both signaling molecules and proteins directly involved in DNA replication and repair. Limited availability of cellular zinc due to zinc deficiency could result in a loss of activity of these zinc-dependent proteins involved in the maintenance of DNA integrity and may contribute to the development of cancer. Zinc deficiency has also been shown to upregulate expression of the tumour suppressor protein, p53, but impair the DNA binding abilities of p53, nuclear factor κB (NFκB), and AP-1 transcription factors in rat glioma C6 cells^21^. These studies suggest that a decrease in cellular zinc alone causes DNA damage and impairs DNA damage response mechanisms, resulting in a loss of DNA integrity and potential for increased cancer risk.

There is evidence to suggest an intriguing link between zinc and cancer. In *in vivo* studies, it has been shown that zinc treatment increases resistance against tumour challenge in mice^22^ and decrease the incidence of spontaneous lung tumours arising in mice^23^. Studies from our laboratory have also advocated the inhibitory effects of zinc on the histological changes and antioxidant status in the colon of the rats during the initiation and promotion phase of experimentally induced colon carcinogenesis^24^,^25^. The studies clearly indicated that the administration of zinc in the presence of procarcinogen 1, 2 dimehtylhydrazine (DMH) brings about decrease in tumour incidence and tumour burden as well as profound alterations in the antioxidant status with restoration of normal colonic histoarchitecture. A recent study by our group has demonstrated the regulatory role of zinc on the membrane fluidity parameters and surface abnormalities following colon specific carcinogen treatment to rats^26^.

Zinc has been ascribed roles in the metabolic functions and interaction of malignant cells^27^. The zinc content of leukaemic leukocytes has been found to be reduced^28^, and it has also been reported that zinc deficiency enhances the carcinogenic effects of nitrosomethylbenzylamine^28^. Zowezak *et al*^29^ found an increase in serum copper/zinc ratios in patients with cancers of the lung, breast, gastrointestinal tract and gyneocological malignancy. Nutritional zinc deficiency in rats increases oesophageal cell proliferation and the incidence of N-nitrosomethylbezylamine (NMBA) induced oesophageal tumours. Replenishing zinc with a zinc sufficient diet reduces these effects in zinc deficient rats. Zinc replenishment rapidly induces apoptosis in oesophageal epithelial cells and thereby substantially reduces the development of esophageal cancer^30^. Using the zinc deficient rat model, Fong *et al*^31^ have shown that after a single, otherwise nontumourigenic dose of NMBA^32^, sustained, increased cell proliferation in the zinc deficient oesophageal epithelium was associated with a highly tumourigenic response and accompanying genetic events. In addition, they demonstrated^31^ that, if a zinc-sufficient diet was administered to zinc deficient rats after the second of six NMBA doses, oesophageal cell proliferation was effectively reversed and tumour incidence was reduced from 100 per cent in zinc deficient rats to 14 per cent in pair-fed zinc-replenished rats (whose food consumption matched that of zinc deficient rats) and to 26 per cent in zinc-replenished rats fed a zinc-sufficient diet *ad libitum*^31^. Zinc deficiency in humans is associated with an increased risk of developing esophageal squamous cell carcinoma^33^. Another mechanism by which zinc might prevent cancer is through its effect on angiogenesis and tumour progression. Endostatin is a potent angiogenesis inhibitor both *in vitro* and *in vivo* and has ability to bind Zn2^+^, essential for its antiangiogenic activity^34^. A report by Jaiswal and Narayan^35^ has also stated the mechanisms by which zinc causes growth arrest in colon cancer cells. The results suggest that zinc treatment stabilizes the levels of the wild-type adenomatous polyposis coli (APC) protein at the post-translational level since the APC mRNA levels and the promoter activity of the APC gene were decreased in HCT-116 cells (which express the wild-type APC gene) after treatment with zinc chloride^35^. It has been seen that the most consistent and persistent biochemical characteristic of prostate cancer is the marked decrease in zinc levels in the malignant cells, thus providing compelling evidence that the lost ability of the malignant cells to accumulate zinc is an important factor in the development and progression of prostate malignancy^36^. A report by Gallus *et al*^37^ found a direct association between high zinc intake and prostate cancer risk, particularly for advanced cancers. To sum up, Ho *et al*^38^ proposed the possible mechanisms of zinc chemoprevention which include the effects of zinc on the inhibition of terminal oxidation, induction of mitochondrial apoptogenesis and suppression of NF-κB activity. Zinc may also play an important role in the maintenance of DNA integrity in normal prostate epithelial cells by modulating DNA repair and damage response proteins, especially p53^39^. In addition, findings support the role of zinc transporters as tumour suppressors in the prostate^40^. Therefore, this relationship provides a rational basis for the concept that restoration of high zinc levels in malignant cells could be efficacious in the treatment and prevention of cancer.

Though the results from these studies have suggested the anticancer role of zinc, little is known about the mechanisms by which zinc exerts its action on cancer cells. In particular, it is not known whether zinc directly acts on tumour cells or its *in vivo* action is related to a modulation of the immune effectiveness or to the zinc dependent regulation of the production of other anticancer substances. Some recent fragmentary evidence has indirectly supported the possible modulation of apoptosis by zinc^41^,^42^. In fact, a zinc dependent modulation of reactive oxygen species (ROS), which has been implicated as modulators of apoptosis^41^–^44^, suggests a possible influence of zinc on apoptosis through the modulation of the intracellular redox state.

## Zinc and apoptosis

The maintenance of discrete subcellular pools of zinc is critical for the functional and structural integrity of cells. Among the important biological processes influenced by Zn is apoptosis, a process that is important in cellular homeostasis^45^. It has also been identified as a major mechanism contributing to cell death in response to toxins and in disease, offering hope that novel therapies that target apoptotic pathways may be developed. Zinc becomes cytotoxic if its extracellular concentration exceeds the capacity of the Zn homeostatic system. Elevated extracellular Zn concentrations lead to the breakdown of the Zn transporting system of the plasma membrane. The resulting enhanced intracellular Zn concentration activates the apoptosis^46^.

Apoptosis is a major mechanism of programmed cell death involved in several biological events during tissue development, remodelling or involution. It is a regulated biological mechanism required for the removal and deletion of superfluous, mutant or moderately damaged cells in response to toxic agents^47^. Rather than the cellular ‘homicide’ that occurs in necrotic cell death, apoptosis is a pathway of cellular ‘suicide’. Apoptosis is morphologically distinct from cell death due to lysosomal breakdown and/or necrosis^48^. Apoptosis occurs in two phases: in the first, the biochemical signaling pathways commit a cell to apoptosis and in the second, the executional phase is characterized by morphological changes leading to cell death^5^.

The precise mechanisms underlying the triggering of apoptosis are not clear but damage to DNA and activation of the p53 gene appears to be an important component of the process as also is activation of certain members of the caspase family of proteases. Zinc is involved to some extent in both processes^49^. The specific DNA-binding domain of p53 has a complex tertiary structure that is stabilized by Zn^50^. Modulation of binding of p53 to DNA by physiological concentrations of Zn might represent a pathway that regulates p53 activity *in vivo*^51^.

*Zinc in p53 structural stabilization and functional activation*: Zinc has been shown to be responsible for the functional conformation of the tumour suppressor p53 protein^52^ and the addition of the physiologic zinc concentrations was seen to mediate the renaturation of wild type p53^53^. The function of p53 is essential for the maintenance of the nontumourogenic phenotype of cells. It has been shown that p53, in its conformational wild type, induces cell cycle arrest in response to DNA damage and that mutations in p53 eliminate this response resulting in an enhanced frequency of genomic rearrangements. The data reported by Provinciali *et al*^54^, clearly demonstrate that zinc induces a transcriptional activation of the p53 gene, with increased expression of p53 mRNA and protein, which correlates with the induction of apoptosis. Besides this effect, zinc might also act on the conformation of the p53 protein of the tumour cells. It has been demonstrated that p53 contains a tightly bound zinc atom that is necessary for the DNA binding activity of the protein^52^. Further, metal chelating reagents results in a reversible conformational alteration of p53 (from wild type to mutant), consistent with zinc playing a critical structural role of p53 protein. On the basis of these findings, the effect of zinc on cancer cells might also be explained by a conformational change of p53 protein, thus restoring its biological activity *i.e*., the tumour suppressor effect of protein and stopping the progression of cancer cell mitosis determining cell death.

*Zinc in caspase activation*: Caspase-6 is the most sensitive apoptosis-related molecular target of Zn. It cleaves and activates the proenzyme form of caspase-3 and is also responsible for the cleavage of lamins and therefore, is directly involved in nuclear membrane dissolution^5^. The balance between life and cell death is maintained by several Zn channels, controlling the intracellular Zn movements and the free amount of the metal^55^. It has been shown that Zn supplementation in old mice is capable to restore immune efficiency with no interference on already high metallothioneins (MTs) levels^56^. As such, more Zn ions bioavailability occurs with subsequent extension of the maximum life span due to significant reductions of deaths from infections^57^. Thus, supplementing Zn might induce MTs to regain their role of protection and it may arrest tumour growth and lead cancer cells to cell death by means of p21 overexpression^58^. This phenomenon has also been suggested for increased α-2 macroglobulin concentrations in cervical carcinoma^59^. Zinc induces apoptosis via deficiency as well as overload. Deprivation of zinc by chelation in various mammalian cell types induced programmed cell death^60^. In C6 cells, complexation of zinc by the membrane-permeable zinc chelator *N,N,N´,N´*-terakis-(2-pyridylmethyl) ethylenediamine (TPEN) evoked internucleosomal DNA fragmentation^61^, whereas addition of 10-50 µM ZnCl_2_ protected the cells against DNA fragmentation. However, zinc at elevated concentrations also induces apoptosis with effects similar to those caused by cadmium. Zinc concentrations of 150 µM and above induced apoptosis in C6 glioma cells that involved breakdown of the mitochondrial membrane potential, chromatin condensation and nuclear fragmentation, and internucleosomal DNA fragmentation^61^.

## Human epidemiological/clinical studies with zinc

The role of zinc in cancer has received increasing attention as a link between zinc deficiency and cancer has now been established in human studies. It is now reported that zinc status is compromised in cancer patients compared to healthy people. Abnet *et al*^33^ observed an initial connection between zinc and oesophageal squamous cell carcinoma in humans and their findings clearly demonstrated significantly lower average tissue zinc concentration in subjects who developed oesophageal cancer than in control subjects. Lee *et al*^62^ also reported that intake of dietary zinc is associated with a decreased risk of both proximal and distal colon cancer in postmenopausal women. Further, a report by Prasad *et al*^63^ also provides evidence based on zinc deficiency and cell mediated immune disorders, and the effects of zinc status on clinical morbidities in patients with head and neck cancer. Zinc deficiency and cell mediated immune dysfunctions were frequently present in patients with head and neck cancer and zinc deficiency was associated with an increased tumour size and the overall stage of the cancer^63^. Abdulla *et al*^64^ observed that plasma zinc was decreased and the copper:zinc ratio in the plasma was significantly higher in patients with squamous cell carcinoma of the head and neck in comparison to healthy controls. Also, the role of zinc in the development and progression of prostate malignancy and its potential application in the prevention and treatment of prostate cancer are known^65^. The overwhelming clinical evidence provides a compelling rational basis for the concept that prostate zinc accumulation is an important factor in the development and progression of prostate malignancy; and that zinc could be efficacious in the prevention and treatment of prostate cancer^65^.

## Zinc: Future Prospects

It is clear from literature that zinc is of extraordinary and diverse importance in cancer biology. Despite encouraging recent progress about the role of zinc in cancer, research is still at a relatively early stage of its evolution. Accelerated research is required to achieve a clear understanding with regard to other definitive bioassays including protein expression and documentation of specific molecular markers as well as zinc homeostatic mechanism in immune system and signal transduction to establish the exact mechanism for zinc-mediated cancer chemoprevention. Only by understanding the basic mechanisms by which zinc can exert its chemopreventive properties we would be able to devise rational uses for this metal as an intervention in cancer management.
